# Supplementary material for: Strong biomechanical relationships bias the tempo and mode of morphological evolution
Source: eLife. 2018 Aug 9;7:e37621. doi: 10.7554/eLife.37621 (PMC6133543; doi:10.7554/eLife.37621)
Supplement: Supplementary file 1. — For each trait (KT, input link, output link, and coupler link), the Brownian Motion evolutionary rate parameter (σ2) is given. For each comparison, the AICC score for a model in which rates are allowed to vary (obs.) and constrained to be equal (const.) are given, as are the Likelihood Ratio Test (LRT) score and corresponding p value for df=1. [file elife-37621-supp1.docx]

**Supplementary File 1.** Pair-wise comparisons of evolutionary rate. For each trait (KT, input link, output link, and coupler link), the Brownian Motion evolutionary rate parameter (σ^2^) is given. For each comparison, the AIC_C_ score for a model in which rates are allowed to vary (obs.) and constrained to be equal (const.) are given, as are the Likelihood Ratio Test (LRT) score and corresponding *p* value for df=1.

**Cichlids**

| Evolutionary rate, σ^2^ | KT | | Input | Output | Coupler |
| --- | --- | --- | --- | --- | --- |
|  | | 5.3e-4 | 7.9e-4 | 2.7e-4 | 4.0e-4 |
| Comparison | | AIC_observed_ | AIC_constrained_ | LRT | *p* |
| Input - Output | | -162.627 | -155.102 | 9.525 | 0.002 |
| Input - Coupler | | -156.213 | -153.542 | 4.671 | 0.031 |
| Output - Coupler | | -178.536 | -179.343 | 1.194 | 0.275 |

**Mantis Shrimp**

| Evolutionary rate, σ^2^ | KT | | Input | Output | Coupler |
| --- | --- | --- | --- | --- | --- |
|  | | 1.2e-4 | 3.8e-5 | 1.7e-4 | 2.2e-5 |
| Comparison | | AIC_observed_ | AIC_constrained_ | LRT | *p* |
| Input - Output | | -206.227 | -189.302 | 18.925 | 1.4e-5 |
| Input - Coupler | | -383.040 | -383.936 | 2.897 | 0.089 |
| Output - Coupler | | -227.024 | -195.197 | 33.827 | 6.0e-9 |

**Sunfish**

| Evolutionary rate, σ^2^ | KT | | Input | Output | Coupler |
| --- | --- | --- | --- | --- | --- |
|  | | 6.9e-4 | 3.7e-5 | 4.8e-4 | 1.0e-4 |
| Comparison | | AIC_observed_ | AIC_constrained_ | LRT | *p* |
| Input - Output | | -125.984 | -93.906 | 34.078 | 5.3e-9 |
| Input - Coupler | | -145.052 | -142.272 | 4.779 | 0.029 |
| Output - Coupler | | -95.769 | -87.164 | 10.604 | 0.001 |

**Wrasses**

| Evolutionary rate, σ^2^ | KT | | Input | Output | Coupler |
| --- | --- | --- | --- | --- | --- |
|  | | 5.5e-3 | 1.9e-3 | 3.1e-3 | 1.5e-3 |
| Comparison | | AIC_observed_ | AIC_constrained_ | LRT | *p* |
| Input - Output | | -413.880 | -417.389 | 5.509 | 0.019 |
| Input - Coupler | | -346.797 | -346.405 | 1.608 | 0.205 |
| Output - Coupler | | -553.817 | -537.762 | 18.056 | 2.1e-5 |
