# Supplementary material for: Strong biomechanical relationships bias the tempo and mode of morphological evolution
Source: eLife. 2018 Aug 9;7:e37621. doi: 10.7554/eLife.37621 (PMC6133543; doi:10.7554/eLife.37621)
Supplement: Supplementary file 2. — Therefore, for these systems, we repeated pair-wise comparisons of evolutionary rate while incorporating intraspecific measurement error (With Error). We also present results for rate comparisons without measurement error incorporated (No Error). For each comparison, the AIC score for a model in which rates are allowed to vary (AICobserved) and constrained to be equal (AICconstrained) are given, as are the Likelihood Ratio Test (LRT) score and corresponding p value for df=1. Evolutionary rate differences between linkages were robust to measurement error. [file elife-37621-supp2.docx]

**Supplementary File 2.** Variance was substantially higher for the output link in mantis shrimp and sunfish. Therefore, for these systems, we repeated pair-wise comparisons of evolutionary rate while incorporating intraspecific measurement error (With Error). We also present results for rate comparisons without measurement error incorporated (No Error). For each comparison, the AIC score for a model in which rates are allowed to vary (AIC_observed_) and constrained to be equal (AIC_constrained_) are given, as are the Likelihood Ratio Test (LRT) score and corresponding *p* value for df=1. Evolutionary rate differences between linkages were robust to measurement error.

**Mantis Shrimp**

| Comparison | AIC_observed_ | AIC_constrained_ | LRT | *p* |
| --- | --- | --- | --- | --- |
| Input – Output (No Error) | -206.227 | -189.302 | 18.925 | 1.4e-5 |
| Input – Output (With Error) | -193.252 | -187.622 | 7.631 | 0.006 |
| Output – Coupler (No Error) | -227.024 | -195.197 | 33.827 | 6.0e-9 |
| Output – Coupler (With Error) | -207.064 | -192.688 | 16.376 | 5.2e-5 |

**Sunfish**

| Comparison | AIC_observed_ | AIC_constrained_ | LRT | *p* |
| --- | --- | --- | --- | --- |
| Input – Output (No Error) | -125.984 | -93.906 | 34.078 | 5.3e-9 |
| Input – Output (With Error) | -107.718 | -85.197 | 24.521 | 7.4e-7 |
| Output – Coupler (No Error) | -95.769 | -87.164 | 10.604 | 0.001 |
| Output – Coupler (With Error) | -89.443 | -82.770 | 8.763 | 0.003 |
