# Supplementary material for: Strong biomechanical relationships bias the tempo and mode of morphological evolution
Source: eLife. 2018 Aug 9;7:e37621. doi: 10.7554/eLife.37621 (PMC6133543; doi:10.7554/eLife.37621)
Supplement: Supplementary file 3. — KT is inversely correlated with the input link and, to a lesser extent, positively correlated with the coupler link. To view these relationships, scroll the plot to place the input and coupler links as x and y-axes, and note the linear correlation between the two variables, coupled with a distinct color transition in the KT color map overlaid on the data. To zoom in and out of the plot scroll upwards and downwards, respectively. [file elife-37621-supp3.zip › elife-37621-supp3-v2.html]

##

##
