# Supplementary material for: Strong biomechanical relationships bias the tempo and mode of morphological evolution
Source: eLife. 2018 Aug 9;7:e37621. doi: 10.7554/eLife.37621 (PMC6133543; doi:10.7554/eLife.37621)
Supplement: Supplementary file 7. — For each shift, the branch and the shift’s posterior probability (pp) are given. The branches that were strongly supported (pp > 0.5) are denoted in bold, and depicted in Figure 3. [file elife-37621-supp7.docx]

**Supplementary File 7.** Analysis of evolutionary shifts for one mechanical trait (KT) and three morphological traits (input link, output link, and coupler link) in wrasses. For each shift, the branch and the shift’s posterior probability (pp) are given. The branches that were strongly supported (pp > 0.5) are denoted in bold, and depicted in Figure 3.

| Trait | Branch Number | pp |  | Trait | Branch Number | pp |
| --- | --- | --- | --- | --- | --- | --- |
| KT | **185** | **0.99** |  | Output | **142** | **0.88** |
|  | **59** | **0.99** |  |  | **185** | **0.77** |
|  | **142**  196  39  197  143  125  43  152  150  127  167  32  17  37  38  65 | **0.67**  0.48  0.41  0.37  0.30  0.22  0.16  0.15  0.15  0.14  0.14  0.13  0.12  0.12  0.12  0.11 |  |  | 47  20  59  60  125  74  56  183  8  143  50  73 | 0.27  0.22  0.21  0.18  0.17  0.14  0.13  0.12  0.12  0.11  0.10  0.10 |
|  |  |  |  | Coupler | **131**  61  185  117 | **0.58**  0.45  0.42  0.41 |
| Input | **185** | **0.97** |  |  | 198 | 0.34 |
|  | **59** | **0.95** |  |  | 97 | 0.26 |
|  | **160** | **0.70** |  |  | 32 | 0.19 |
|  | **197** | **0.62** |  |  | 38 | 0.18 |
|  | 157 | 0.49 |  |  | 34 | 0.14 |
|  | 109 | 0.42 |  |  | 157 | 0.13 |
|  | 37 | 0.36 |  |  |  |  |
|  | 196 | 0.34 |  |  |  |  |
|  | 156 | 0.24 |  |  |  |  |
|  | 17 | 0.18 |  |  |  |  |
|  | 94 | 0.18 |  |  |  |  |
|  | 43 | 0.18 |  |  |  |  |
|  | 62 | 0.18 |  |  |  |  |
|  | 45 | 0.16 |  |  |  |  |
|  | 19 | 0.14 |  |  |  |  |
|  | 158 | 0.13 |  |  |  |  |
|  | 167 | 0.13 |  |  |  |  |
|  | 180 | 0.12 |  |  |  |  |
|  | 63 | 0.12 |  |  |  |  |
|  | 39 | 0.11 |  |  |  |  |
|  | 61 | 0.10 |  |  |  |  |
