# Supplementary figures and images for: Strong biomechanical relationships bias the tempo and mode of morphological evolution
Source: eLife. 2018 Aug 9;7:e37621. doi: 10.7554/eLife.37621 (PMC6133543; doi:10.7554/eLife.37621)

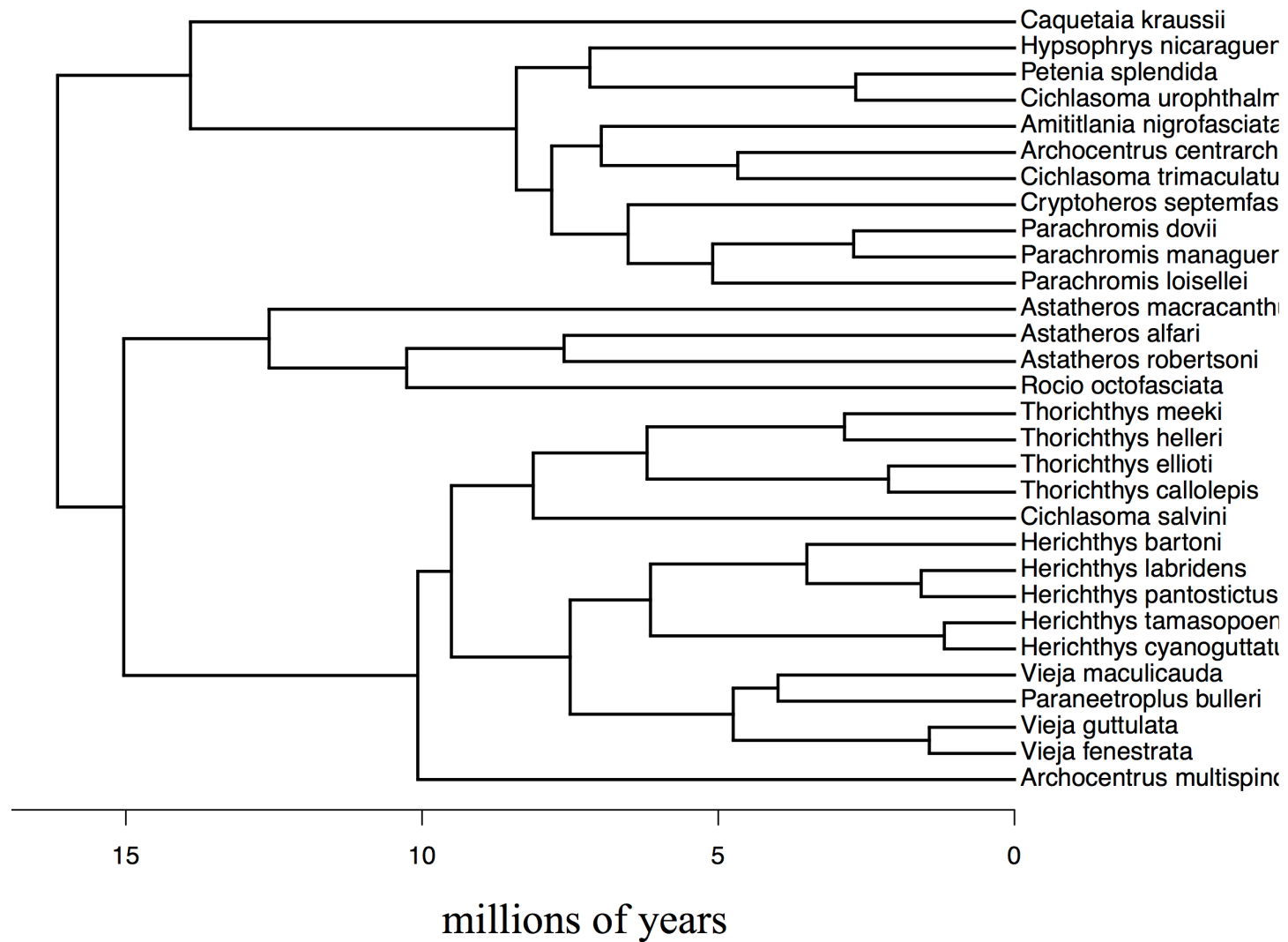

Supplement: Supplementary file 8. — This phylogeny was constructed using the previously-published phylogeny by Hulsey et al., 2010. [file elife-37621-supp8.pdf]

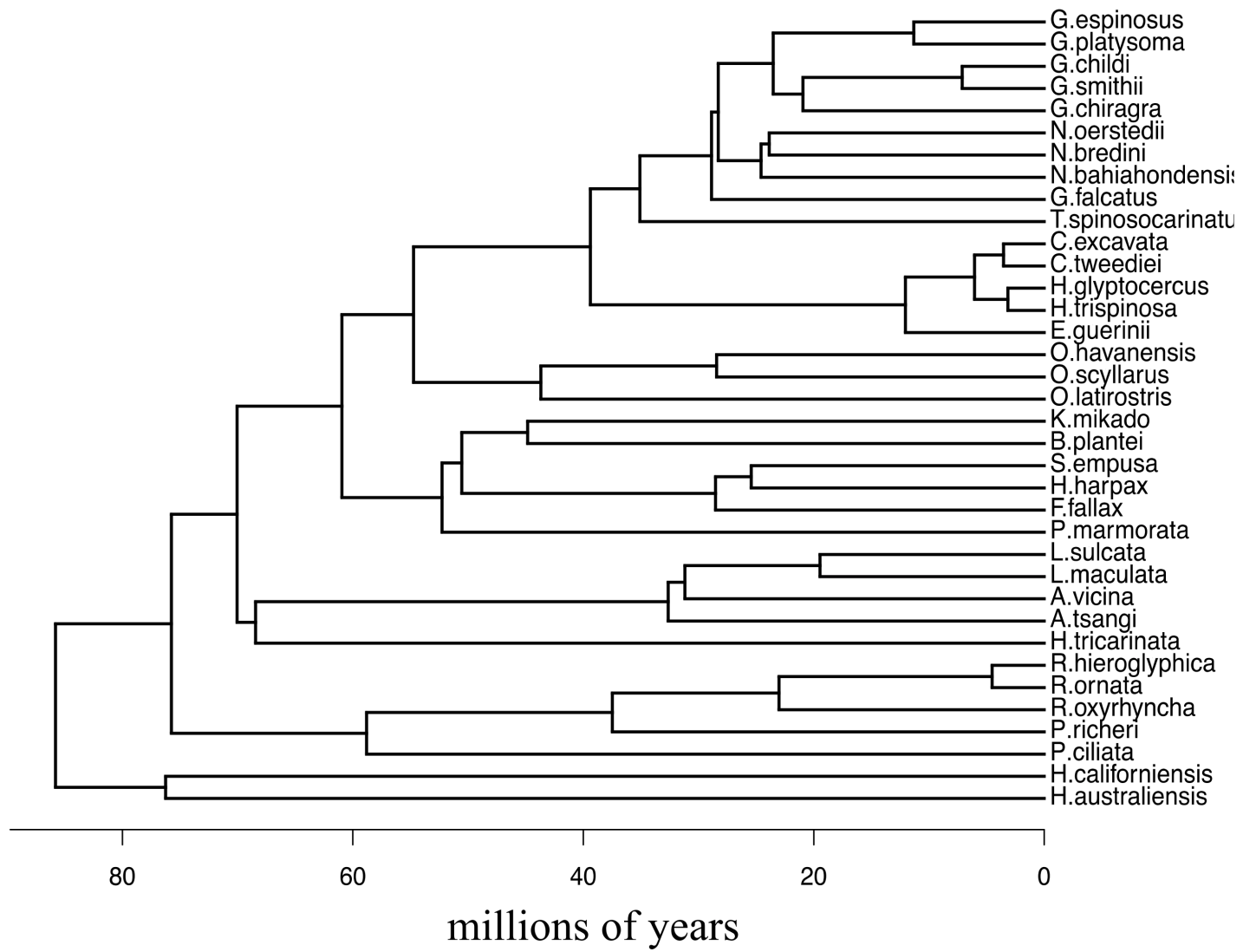

Supplement: Supplementary file 9. [file elife-37621-supp9.pdf]

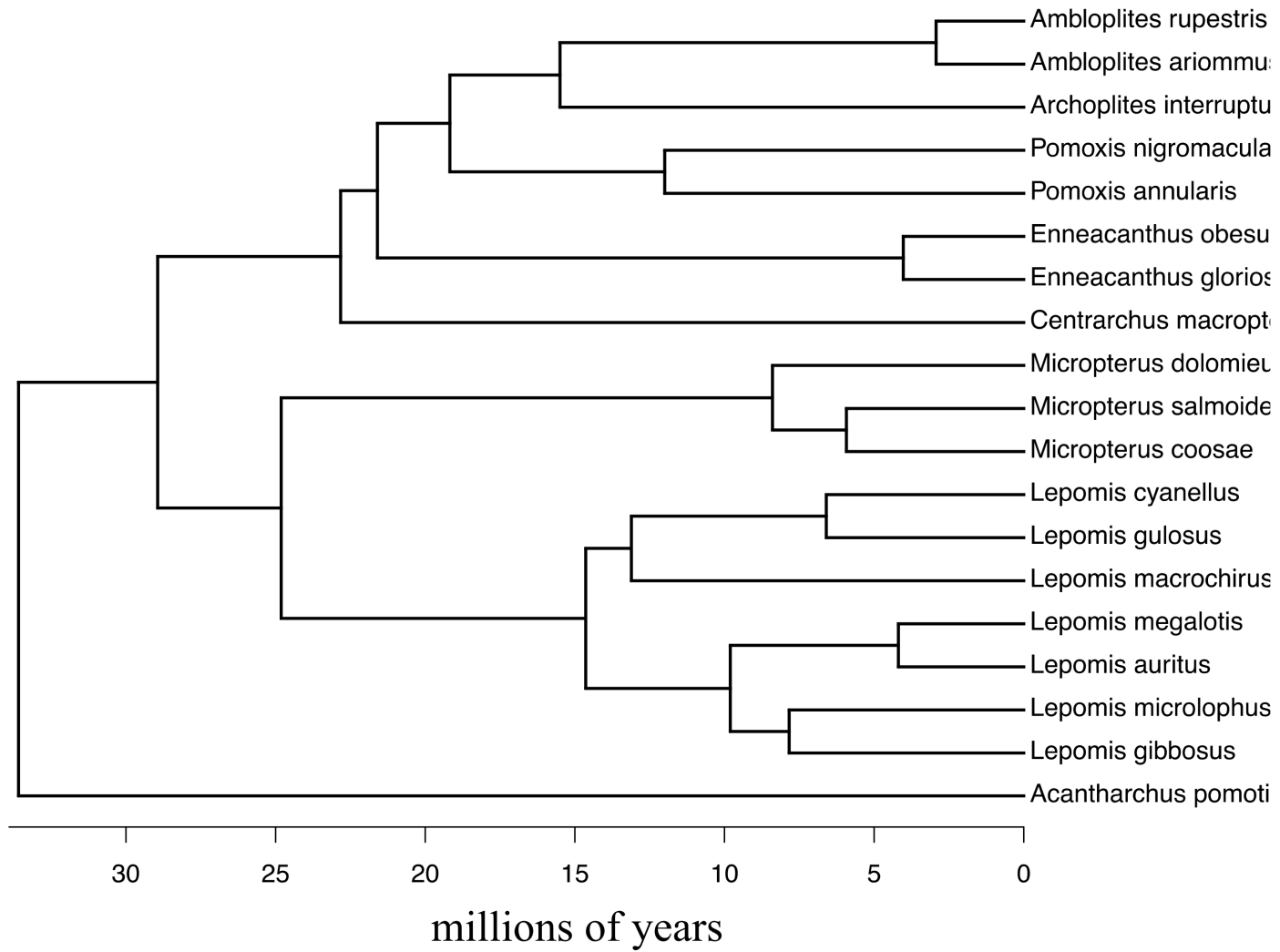

Supplement: Supplementary file 10. — We constructed this phylogeny using the previously-published phylogeny by Near et al., 2005. [file elife-37621-supp10.pdf]

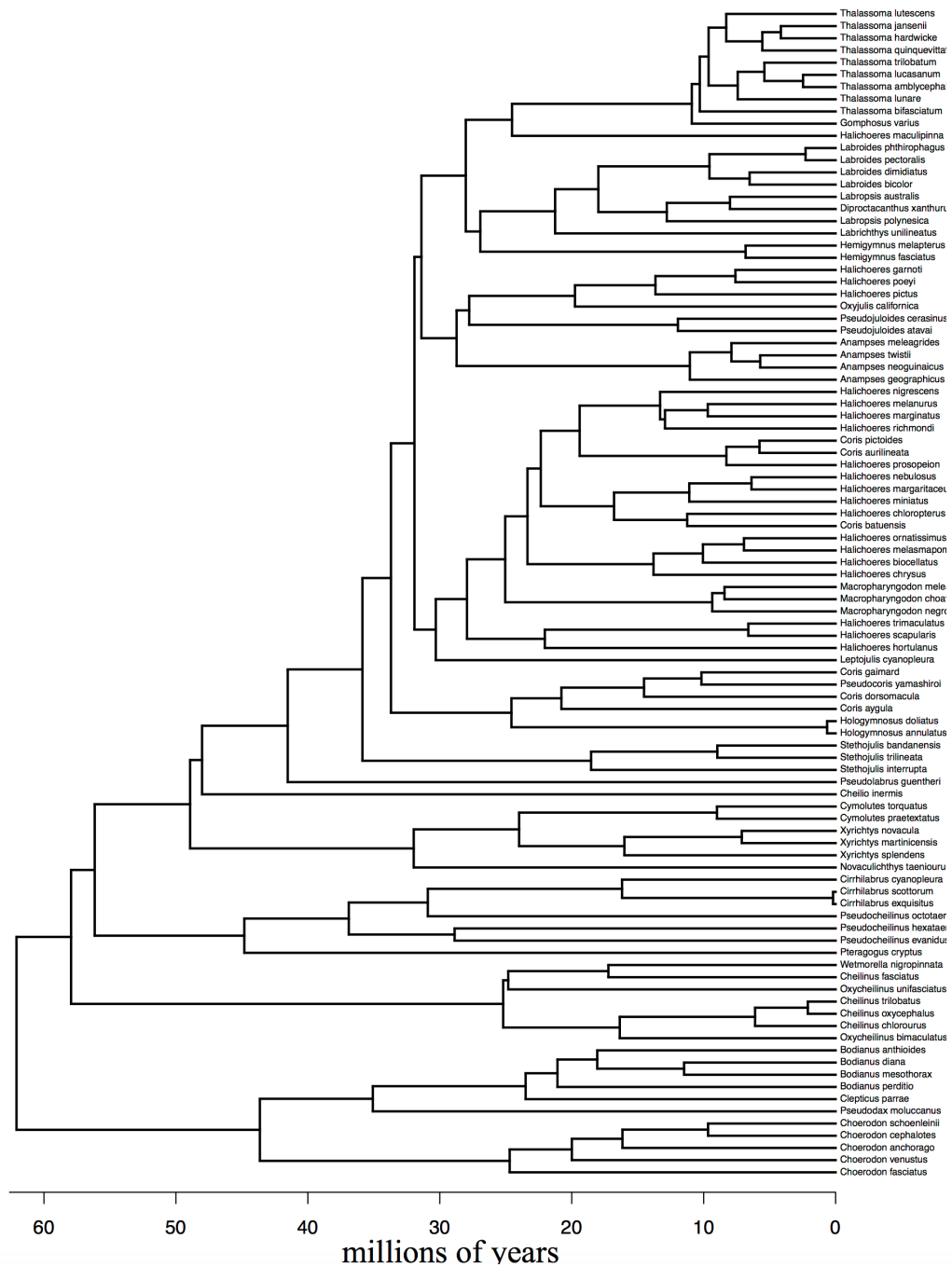

Supplement: Supplementary file 11. — This phylogeny is based on the previously-published wrasse phylogeny by Baliga and Law, 2016. [file elife-37621-supp11.pdf]
