# Supplementary material for: Strong biomechanical relationships bias the tempo and mode of morphological evolution
Source: eLife. 2018 Aug 9;7:e37621. doi: 10.7554/eLife.37621 (PMC6133543; doi:10.7554/eLife.37621)
Supplement: Supplementary file 12. — Data were gathered from Hulsey and Garcia De Leon, 2005. [file elife-37621-supp12.docx]

**Supplementary File 12.** The mechanical and morphological data for the cichlid (Family: Cichlidae) species used in this study. Data were gathered from Hulsey and García de León (2005).

| Species | Input Link | Output Link | Coupler Link | KT |
| --- | --- | --- | --- | --- |
| *Amititlania nigrofasciata* | 0.382 | 0.485 | 0.544 | 0.78 |
| *Archocentrus centrarchus* | 0.455 | 0.535 | 0.455 | 0.83 |
| *Archocentrus multispinosus* | 0.356 | 0.622 | 0.533 | 0.65 |
| *Astatheros alfari* | 0.371 | 0.521 | 0.536 | 0.71 |
| *Astatheros macracanthus* | 0.369 | 0.592 | 0.553 | 0.62 |
| *Astatheros robertsoni* | 0.403 | 0.521 | 0.555 | 0.78 |
| *Caquetaia kraussii* | 0.508 | 0.515 | 0.354 | 0.96 |
| *Cichlasoma salvini* | 0.479 | 0.531 | 0.448 | 0.9 |
| *Cichlasoma trimaculatum* | 0.471 | 0.529 | 0.461 | 0.83 |
| *Cichlasoma urophthalmus* | 0.374 | 0.565 | 0.513 | 0.68 |
| *Cryptoheros septemfasciatus* | 0.356 | 0.438 | 0.562 | 0.7 |
| *Herichthys bartoni* | 0.427 | 0.613 | 0.524 | 0.68 |
| *Herichthys cyanoguttatus* | 0.397 | 0.544 | 0.507 | 0.73 |
| *Herichthys labridens* | 0.387 | 0.495 | 0.523 | 0.79 |
| *Herichthys pantostictus* | 0.405 | 0.532 | 0.516 | 0.76 |
| *Herichthys tamasopoensis* | 0.315 | 0.546 | 0.500 | 0.58 |
| *Hypsophrys nicaraguensis* | 0.347 | 0.460 | 0.621 | 0.75 |
| *Parachromis dovii* | 0.490 | 0.590 | 0.400 | 0.78 |
| *Parachromis loisellei* | 0.551 | 0.641 | 0.449 | 0.84 |
| *Parachromis managuensis* | 0.459 | 0.557 | 0.369 | 0.79 |
| *Paraneetroplus bulleri* | 0.376 | 0.505 | 0.594 | 0.74 |
| *Petenia splendida* | 0.614 | 0.526 | 0.392 | 1.06 |
| *Rocio octofasciata* | 0.381 | 0.557 | 0.443 | 0.71 |
| *Thorichthys callolepis* | 0.392 | 0.474 | 0.567 | 0.83 |
| *Thorichthys ellioti* | 0.367 | 0.469 | 0.531 | 0.76 |
| *Thorichthys helleri* | 0.431 | 0.549 | 0.549 | 0.79 |
| *Thorichthys meeki* | 0.359 | 0.489 | 0.598 | 0.73 |
| *Vieja fenestrata* | 0.394 | 0.551 | 0.520 | 0.73 |
| *Vieja guttulata* | 0.339 | 0.461 | 0.478 | 0.76 |
| *Vieja maculicauda* | 0.357 | 0.500 | 0.536 | 0.73 |
