# Supplementary material for: Strong biomechanical relationships bias the tempo and mode of morphological evolution
Source: eLife. 2018 Aug 9;7:e37621. doi: 10.7554/eLife.37621 (PMC6133543; doi:10.7554/eLife.37621)
Supplement: Supplementary file 13. — Data were gathered from Anderson and Patek, 2015. [file elife-37621-supp13.docx]

**Supplementary File 13.** The mechanical and morphological data for the stomatopod species used in this study. Data were gathered from Anderson and Patek (2015).

| Species | Input Link | Output Link | Coupler Link | KT |
| --- | --- | --- | --- | --- |
| *Alachosquilla vicina* | 0.838 | 0.108 | 1.063 | 6.85 |
| *Austrosquilla tsangi* | 0.790 | 0.076 | 1.012 | 9.59 |
| *Busquilla plantei* | 0.704 | 0.106 | 0.977 | 6.32 |
| *Chorisquilla excavata* | 0.632 | 0.134 | 1.015 | 4.92 |
| *Chorisquilla tweedei* | 0.694 | 0.117 | 1.070 | 5.52 |
| *Echinosquilla guerinii* | 0.690 | 0.124 | 1.089 | 4.96 |
| *Fallosquilla fallax* | 0.734 | 0.103 | 1.101 | 6.34 |
| *Gonodactylaceus falcatus* | 0.660 | 0.109 | 1.041 | 5.61 |
| *Gonodactylellus espinosus* | 0.648 | 0.116 | 1.188 | 4.82 |
| *Gonodactylus childi* | 0.606 | 0.086 | 1.151 | 6.21 |
| *Gonodactylus chiragra* | 0.686 | 0.118 | 0.998 | 5.51 |
| *Gonodactylus platysoma* | 0.663 | 0.118 | 1.076 | 5.16 |
| *Gonodactylus smithii* | 0.685 | 0.128 | 1.112 | 4.74 |
| *Haptosquilla glyptocercus* | 0.623 | 0.110 | 1.019 | 5.4 |
| *Haptosquilla trispinosa* | 0.661 | 0.105 | 1.011 | 6.01 |
| *Harpiosquilla harpax* | 0.859 | 0.095 | 1.207 | 7.62 |
| *Hemisquilla australiensis* | 0.827 | 0.131 | 0.957 | 5.86 |
| *Hemisquilla californiensis* | 0.887 | 0.124 | 0.952 | 6.45 |
| *Heterosquilla tricarinata* | 0.805 | 0.082 | 0.958 | 9.99 |
| *Kempina mikado* | 0.850 | 0.122 | 1.150 | 5.93 |
| *Lysiosquillina maculata* | 0.701 | 0.061 | 1.157 | 9.98 |
| *Lysiosquillina sulcata* | 0.765 | 0.077 | 1.129 | 8.73 |
| *Neogonodactylus bahiahondensis* | 0.594 | 0.107 | 1.151 | 5.11 |
| *Neogonodactylus bredini* | 0.645 | 0.098 | 1.046 | 5.74 |
| *Neogonodactylus oerstedii* | 0.688 | 0.099 | 1.092 | 6.37 |
| *Odontodactylus havanensis* | 0.734 | 0.115 | 1.079 | 5.77 |
| *Odontodactylus latirostris* | 0.781 | 0.129 | 1.090 | 5.45 |
| *Odontodactylus scyllarus* | 0.778 | 0.128 | 1.065 | 5.44 |
| *Pseudosquilla ciliata* | 0.773 | 0.100 | 1.056 | 7.01 |
| *Pseudosquillana richeri* | 0.978 | 0.087 | 1.318 | 8.68 |
| *Pseudosquillopsis marmorata* | 0.725 | 0.107 | 1.058 | 6.39 |
| *Raoulserenea hieroglyphica* | 0.825 | 0.095 | 1.061 | 8.38 |
| *Raoulserenea ornata* | 0.781 | 0.103 | 1.056 | 8.64 |
| *Raoulserenea oxyrhyncha* | 0.811 | 0.081 | 1.051 | 8.99 |
| *Squilla empusa* | 0.794 | 0.118 | 1.132 | 5.9 |
| *Taku spinosocarinatus* | 0.647 | 0.129 | 1.094 | 4.56 |
