# Supplementary material for: Strong biomechanical relationships bias the tempo and mode of morphological evolution
Source: eLife. 2018 Aug 9;7:e37621. doi: 10.7554/eLife.37621 (PMC6133543; doi:10.7554/eLife.37621)
Supplement: Supplementary file 14. — Data were gathered from Hu et al., 2017. [file elife-37621-supp14.docx]

**Supplementary File 14.** The mechanical and morphological data for the sunfish (Family: Centrarchidae) species used in this study. Data were gathered from Hu et al. (2017).

| Species | Input Link | Output Link | Coupler Link | KT |
| --- | --- | --- | --- | --- |
| *Micropterus coosae* | 0.852 | 0.158 | 0.873 | 5.299 |
| *Pomoxis annularis* | 0.914 | 0.110 | 0.820 | 8.103 |
| *Micropterus dolomieu* | 0.893 | 0.113 | 0.771 | 8.231 |
| *Pomoxis nigromaculatus* | 0.941 | 0.112 | 0.731 | 8.393 |
| *Ambloplites ariommus* | 0.929 | 0.109 | 0.593 | 8.512 |
| *Micropterus salmoides* | 0.922 | 0.096 | 0.754 | 9.401 |
| *Enneacanthus obesus* | 1.007 | 0.096 | 0.707 | 10.283 |
| *Archoplites interruptus* | 0.965 | 0.086 | 0.746 | 10.937 |
| *Lepomis cyanellus* | 1.034 | 0.092 | 0.737 | 10.980 |
| *Enneacanthus gloriosus* | 1.055 | 0.090 | 0.743 | 11.237 |
| *Lepomis macrochirus* | 1.029 | 0.084 | 0.735 | 11.819 |
| *Lepomis gulosus* | 0.971 | 0.083 | 0.683 | 11.962 |
| *Ambloplites rupestris* | 0.998 | 0.083 | 0.688 | 12.069 |
| *Lepomis auritus* | 0.936 | 0.073 | 0.706 | 12.604 |
| *Acantharchus pomotis* | 1.010 | 0.074 | 0.684 | 13.208 |
| *Lepomis megalotis* | 0.947 | 0.074 | 0.679 | 13.284 |
| *Centrarchus macropterus* | 1.040 | 0.075 | 0.726 | 13.371 |
| *Lepomis microlophus* | 1.044 | 0.074 | 0.710 | 14.066 |
| *Lepomis gibbosus* | 0.983 | 0.067 | 0.788 | 14.096 |
