# Supplementary material for: Strong biomechanical relationships bias the tempo and mode of morphological evolution
Source: eLife. 2018 Aug 9;7:e37621. doi: 10.7554/eLife.37621 (PMC6133543; doi:10.7554/eLife.37621)
Supplement: Supplementary file 15. — Data were gathered from Alfaro et al., 2005. [file elife-37621-supp15.docx]

**Supplementary File 15.** The mechanical and morphological data for the wrasses (Family: Labridae) used in this study. Data were gathered from Alfaro et al. (2005).

| Species | Input Link | Output Link | Coupler Link | KT |
| --- | --- | --- | --- | --- |
| *Anampses caeruleopunctatus* | 0.37 | 0.64 | 0.64 | 0.47 |
| *Anampses geographicus* | 0.29 | 0.52 | 0.67 | 0.55 |
| *Anampses meleagrides* | 0.34 | 0.6 | 0.64 | 0.54 |
| *Anampses neoguinaicus* | 0.31 | 0.58 | 0.57 | 0.47 |
| *Anampses twistii* | 0.32 | 0.49 | 0.64 | 0.64 |
| *Bodianus anthioides* | 0.4 | 0.57 | 0.36 | 0.7 |
| *Bodianus axillaris* | 0.43 | 0.51 | 0.43 | 0.87 |
| *Bodianus diana* | 0.5 | 0.52 | 0.47 | 0.9 |
| *Bodianus mesothorax* | 0.44 | 0.56 | 0.38 | 0.75 |
| *Bodianus perditio* | 0.4 | 0.62 | 0.39 | 0.62 |
| *Cheilinus chlorourus* | 0.43 | 0.48 | 0.48 | 0.92 |
| *Cheilinus fasciatus* | 0.42 | 0.56 | 0.5 | 0.75 |
| *Cheilinus oxycephalus* | 0.5 | 0.47 | 0.47 | 1.17 |
| *Cheilinus trilobatus* | 0.39 | 0.51 | 0.48 | 0.79 |
| *Cheilinus undulatus* | 0.37 | 0.5 | 0.61 | 0.75 |
| *Cheilio inermis* | 0.43 | 0.59 | 0.92 | 0.65 |
| *Choerodon anchorago* | 0.31 | 0.53 | 0.48 | 0.59 |
| *Choerodon cephalotes* | 0.31 | 0.56 | 0.57 | 0.54 |
| *Choerodon fasciatus* | 0.37 | 0.49 | 0.43 | 0.8 |
| *Choerodon schoenleinii* | 0.34 | 0.51 | 0.52 | 0.66 |
| *Choerodon venustus* | 0.3 | 0.57 | 0.58 | 0.53 |
| *Cirrhilabrus cyanopleura* | 0.51 | 0.47 | 0.48 | 1.09 |
| *Cirrhilabrus exquisitus* | 0.51 | 0.54 | 0.45 | 0.98 |
| *Cirrhilabrus scottorum* | 0.47 | 0.46 | 0.49 | 1.08 |
| *Clepticus parrae* | 0.68 | 0.36 | 0.61 | 1.95 |
| *Coris aurilineata* | 0.38 | 0.55 | 0.52 | 0.69 |
| *Coris aygula* | 0.38 | 0.59 | 0.49 | 0.64 |
| *Coris batuensis* | 0.36 | 0.51 | 0.47 | 0.67 |
| *Coris dorsomacula* | 0.44 | 0.52 | 0.51 | 0.84 |
| *Coris gaimard* | 0.4 | 0.5 | 0.5 | 0.81 |
| *Coris pictoides* | 0.43 | 0.61 | 0.51 | 0.69 |
| *Cymolutes praetextatus* | 0.4 | 0.38 | 0.55 | 1.26 |
| *Cymolutes torquatus* | 0.41 | 0.4 | 0.52 | 1.03 |
| *Diproctacanthus xanthurus* | 0.4 | 0.53 | 0.59 | 0.76 |
| *Gomphosus varius* | 0.49 | 0.45 | 0.58 | 1.15 |
| *Halichoeres biocellatus* | 0.36 | 0.47 | 0.57 | 0.83 |
| *Halichoeres chloropterus* | 0.4 | 0.53 | 0.48 | 0.73 |
| *Halichoeres chrysus* | 0.41 | 0.54 | 0.58 | 0.73 |
| *Halichoeres garnoti* | 0.38 | 0.49 | 0.58 | 0.78 |
| *Halichoeres hortulanus* | 0.39 | 0.43 | 0.55 | 0.97 |
| *Halichoeres maculipinna* | 0.32 | 0.49 | 0.57 | 0.88 |
| *Halichoeres margaritaceus* | 0.36 | 0.51 | 0.5 | 0.71 |
| *Halichoeres marginatus* | 0.33 | 0.43 | 0.58 | 0.85 |
| *Halichoeres melanurus* | 0.38 | 0.48 | 0.53 | 0.8 |
| *Halichoeres melasmapomus* | 0.45 | 0.49 | 0.52 | 0.9 |
| *Halichoeres miniatus* | 0.42 | 0.53 | 0.48 | 0.79 |
| *Halichoeres nebulosus* | 0.38 | 0.6 | 0.44 | 0.63 |
| *Halichoeres nigrescens* | 0.42 | 0.47 | 0.51 | 0.9 |
| *Halichoeres ornatissimus* | 0.4 | 0.49 | 0.58 | 0.85 |
| *Halichoeres pictus* | 0.5 | 0.43 | 0.61 | 1.2 |
| *Halichoeres poeyi* | 0.39 | 0.46 | 0.58 | 0.84 |
| *Halichoeres prosopeion* | 0.42 | 0.54 | 0.54 | 0.81 |
| *Halichoeres richmondi* | 0.39 | 0.45 | 0.62 | 1.11 |
| *Halichoeres scapularis* | 0.42 | 0.48 | 0.53 | 0.9 |
| *Halichoeres trimaculatus* | 0.41 | 0.49 | 0.53 | 0.86 |
| *Hemigymnus fasciatus* | 0.32 | 0.53 | 0.52 | 0.67 |
| *Hemigymnus melapterus* | 0.33 | 0.53 | 0.52 | 0.6 |
| *Hologymnosus annulatus* | 0.4 | 0.46 | 0.71 | 0.87 |
| *Hologymnosus doliatus* | 0.39 | 0.52 | 0.7 | 0.74 |
| *Labrichthys unilineatus* | 0.37 | 0.54 | 0.57 | 0.72 |
| *Labroides bicolor* | 0.4 | 0.61 | 0.74 | 0.55 |
| *Labroides dimidiatus* | 0.39 | 0.54 | 0.68 | 0.71 |
| *Labroides pectoralis* | 0.38 | 0.59 | 0.7 | 0.56 |
| *Labroides phthirophagus* | 0.36 | 0.44 | 0.68 | 0.79 |
| *Labropsis australis* | 0.35 | 0.56 | 0.59 | 0.6 |
| *Labropsis polynesica* | 0.33 | 0.45 | 0.61 | 0.86 |
| *Leptojulis cyanopleura* | 0.45 | 0.55 | 0.56 | 0.82 |
| *Macropharyngodon choati* | 0.39 | 0.5 | 0.43 | 0.81 |
| *Macropharyngodon meleagris* | 0.32 | 0.48 | 0.44 | 0.67 |
| *Macropharyngodon negrosensis* | 0.39 | 0.49 | 0.45 | 0.84 |
| *Novaculichthys taeniourus* | 0.39 | 0.49 | 0.46 | 0.87 |
| *Oxycheilinus bimaculatus* | 0.41 | 0.55 | 0.49 | 0.75 |
| *Oxycheilinus digramma* | 0.44 | 0.51 | 0.57 | 0.82 |
| *Oxycheilinus unifasciatus* | 0.4 | 0.53 | 0.55 | 0.73 |
| *Oxyjulis californica* | 0.5 | 0.45 | 0.74 | 0.99 |
| *Pseudocheilinus evanidus* | 0.43 | 0.55 | 0.47 | 0.78 |
| *Pseudocheilinus hexataenia* | 0.48 | 0.45 | 0.46 | 1.18 |
| *Pseudocheilinus octotaenia* | 0.49 | 0.55 | 0.49 | 0.83 |
| *Pseudocoris yamashiroi* | 0.53 | 0.57 | 0.54 | 0.92 |
| *Pseudodax moluccanus* | 0.42 | 0.49 | 0.38 | 0.89 |
| *Pseudojuloides atavai* | 0.43 | 0.39 | 0.73 | 1.09 |
| *Pseudojuloides cerasinus* | 0.43 | 0.51 | 0.68 | 0.83 |
| *Pseudolabrus guentheri* | 0.39 | 0.54 | 0.47 | 0.76 |
| *Pteragogus cryptus* | 0.45 | 0.52 | 0.45 | 0.84 |
| *Stethojulis bandanensis* | 0.34 | 0.53 | 0.49 | 0.65 |
| *Stethojulis interrupta* | 0.39 | 0.54 | 0.55 | 0.7 |
| *Stethojulius strigiventer* | 0.44 | 0.64 | 0.6 | 0.55 |
| *Stethojulis trilineata* | 0.37 | 0.46 | 0.54 | 0.81 |
| *Thalassoma amblycephalum* | 0.44 | 0.51 | 0.47 | 0.92 |
| *Thalassoma bifasciatum* | 0.42 | 0.44 | 0.56 | 0.97 |
| *Thalassoma hardwicke* | 0.41 | 0.46 | 0.52 | 0.89 |
| *Thalassoma jansenii* | 0.38 | 0.45 | 0.51 | 0.86 |
| *Thalassoma lucasanum* | 0.4 | 0.41 | 0.55 | 1 |
| *Thalassoma lunare* | 0.35 | 0.47 | 0.44 | 0.82 |
| *Thalassoma lutescens* | 0.36 | 0.51 | 0.52 | 0.72 |
| *Thalassoma quinquevittatum* | 0.39 | 0.47 | 0.53 | 0.87 |
| *Thalassoma trilobatum* | 0.37 | 0.56 | 0.47 | 0.64 |
| *Wetmorella nigropinnata* | 0.57 | 0.48 | 0.57 | 1.11 |
| *Xyrichtys martinicensis* | 0.4 | 0.32 | 0.53 | 1.46 |
| *Xyrichtys novacula* | 0.34 | 0.33 | 0.57 | 1.18 |
| *Xyrichtys splendens* | 0.41 | 0.36 | 0.61 | 1.25 |
