# Supplementary material for: Strong biomechanical relationships bias the tempo and mode of morphological evolution
Source: eLife. 2018 Aug 9;7:e37621. doi: 10.7554/eLife.37621 (PMC6133543; doi:10.7554/eLife.37621)
Supplement: Supplementary file 16. — Table shows PGLS regressions examining the relationship between mobile links (predictor variable) and kinematic transmission (response variable) using static (rather than dynamic) measures of KT in mantis shrimp and sunfish. [file elife-37621-supp16.docx]

**Supplementary File 16.** Analyses of mechanical sensitivity in mantis shrimp and sunfish are robust to static measures of KT (see Methods). Table shows PGLS regressions examining the relationship between mobile links (predictor variable) and kinematic transmission (response variable) using static (rather than dynamic) measures of KT in mantis shrimp and sunfish.

**Mantis Shrimp (df=34)**

| Predictor | Coeff. ± SE | t | *P* | r^2^ |
| --- | --- | --- | --- | --- |
| Input | 0.10 ± 0.28 | 0.36 | 0.720 | -0.025 |
| Output | -0.69 ± 0.10 | -7.26 | 2.1e-8 | 0.597 |
| Coupler | 0.33 ± 0.36 | 0.92 | 0.366 | -0.005 |

**Sunfish (df=18)**

| Predictor | Coeff. ± SE | t | *P* | r^2^ |
| --- | --- | --- | --- | --- |
| Input | 3.0 ± 0.66 | 4.56 | 2.8e-4 | 0.523 |
| Output | -1.20 ± 0.07 | -16.98 | 4.3e-12 | 0.941 |
| Coupler | -0.78 ± 0.63 | -1.25 | 0.229 | 0.030 |
