# Supplementary material for: Strong biomechanical relationships bias the tempo and mode of morphological evolution
Source: eLife. 2018 Aug 9;7:e37621. doi: 10.7554/eLife.37621 (PMC6133543; doi:10.7554/eLife.37621)
Supplement: Supplementary file 17. — Here, the predictor variables were the residuals of mobile link length regressed against fixed link length. Raw data for the wrasses were not available. [file elife-37621-supp17.docx]

**Supplementary File 17.** Using residuals of trait values produces similar mechanical sensitivity results to the commonly utilized size corrections (mobile lever/fixed lever). Here, the predictor variables were the residuals of mobile link length regressed against fixed link length. Raw data for the wrasses were not available.

**Cichlids (df=28)**

| Predictor | Coeff. ± SE | t | *P* | r^2^ |
| --- | --- | --- | --- | --- |
| Input | 0.63 ± 0.09 | 6.77 | <0.001 | 0.607 |
| Output | -0.24 ± 0.24 | 0.24 | 0.328 | -0.001 |
| Coupler | -0.46 ± 0.14 | -3.25 | 0.003 | 0.248 |

**Mantis Shrimp (df=34)**

| Predictor | Coeff. ± SE | t | *P* | r^2^ |
| --- | --- | --- | --- | --- |
| Input | 1.19 ± 0.28 | 4.19 | <0.001 | 0.321 |
| Output | -1.06 ± 0.10 | -11.07 | <0.001 | 0.776 |
| Coupler | 0.07 ± 0.55 | 0.14 | 0.892 | -0.029 |

**Sunfish (df=17)**

| Predictor | Coeff. ± SE | t | *P* | r^2^ |
| --- | --- | --- | --- | --- |
| Input | 3.61 ± 0.67 | 5.35 | <0.001 | 0.619 |
| Output | -1.18 ± 0.05 | -21.80 | <0.001 | 0.965 |
| Coupler | -1.32 ± 0.70 | -1.90 | 0.075 | 0.133 |
